# Adhesive capsulitis following COVID-19 vaccination: a case report and review of literature

**DOI:** 10.1093/jscr/rjad611

**Published:** 2023-11-12

**Authors:** Mohammed Alshehri, Mohammed Alsalman, Firas M Alsebayel

**Affiliations:** Department of Orthopedic Surgery, Ministry of National Guard Health Affairs, Riyadh, Saudi Arabia; Department of Medical Radiology, Ministry of National Guard Health Affairs, Riyadh, Saudi Arabia; Department of Orthopedic Surgery, Ministry of National Guard Health Affairs, Riyadh, Saudi Arabia

**Keywords:** COVID, adhesive capsulitis, SIRVA

## Abstract

Shoulder injury related to vaccine administration (SIRVA) has been frequently reported adverse event following COVID-19 vaccination. Multiple studies have reported various injuries including subacromial bursitis, rotator cuff tears, nerve injury, and most commonly, adhesive capsulitis. Adhesive capsulitis is defined as an inflammatory disease of the joint capsule characterized by pain and stiffness. Herein, we present a case of a 38-year-old female, known to have uncontrolled diabetes mellites and asthma, presented to upper extremity orthopedic clinic complaining of 6 months history of left shoulder pain and limited range of motion following COVID-19 vaccination administration. Clinical examination and radiological studies were consistent with adhesive capsulitis, the patient was then referred for intensive rehabilitation program that provided adequate response. In conclusion, the main etiology of SIRVA has been attributed to suboptimal injection technique, a standardized definition, implementation of safe vaccines injection protocols, and further education and awareness of SIRVA is needed to healthcare practitioners to allow better understanding and prevention.

## Introduction

Shoulder injury related to vaccine administration (SIRVA) has been frequently reported adverse event following COVID-19 vaccination [1, 2]. Multiple studies have reported various injuries including subacromial bursitis, rotator cuff tears, nerve injury, and most commonly, adhesive capsulitis [3]. The exact etiology, pathology, and their relation to COVID-19 vaccination is yet to be determined, however, it has been suggested that improper injection technique could be the cause [4].

Adhesive capsulitis is considered one the most common pathologies in the shoulder region. It’s defined as an inflammatory disease of the joint capsule, its clinical course starts with painful phase characterized by pain and stiffness, which is thought to be caused by fibrous tissue formation around the joint, followed by loss of active and passive range of motion. The exact etiology of adhesive capsulitis is unknown, however, it has been associated with certain systematic diseases such as diabetes and hypothyroidism.

There have been multiple therapeutic regimens for adhesive capsulitis described in the literature. The first line management is physiotherapy. Followed by non-steroidal anti-inflammatory medications. Oral NSAID, steroid injections, and hydrodilatation were also some of the management modalities prior to operative management.

Herein, we present a case of a 38-year-old female, known to have uncontrolled diabetes mellites and asthma, presented to the upper extremity orthopedic clinic complaining of 6 months history of left shoulder pain and limited range of motion. Symptoms started 3 days after receiving COVID-19 vaccination. Symptoms were progressive, aggravated by over the head activity and external rotation. She was right-handed, works as a nurse, and denies any previous history of smoking or alcohol use.

On physical examination, no apparent bruising or swelling was appreciated, nor localized tenderness on palpation. She had active range of motion reaching up to 100° of forward flexion, 80° of abduction, 80° of internal rotation, 20° of external rotation, and 15° of external rotation while the shoulder is abducted.

**Figure 1:**
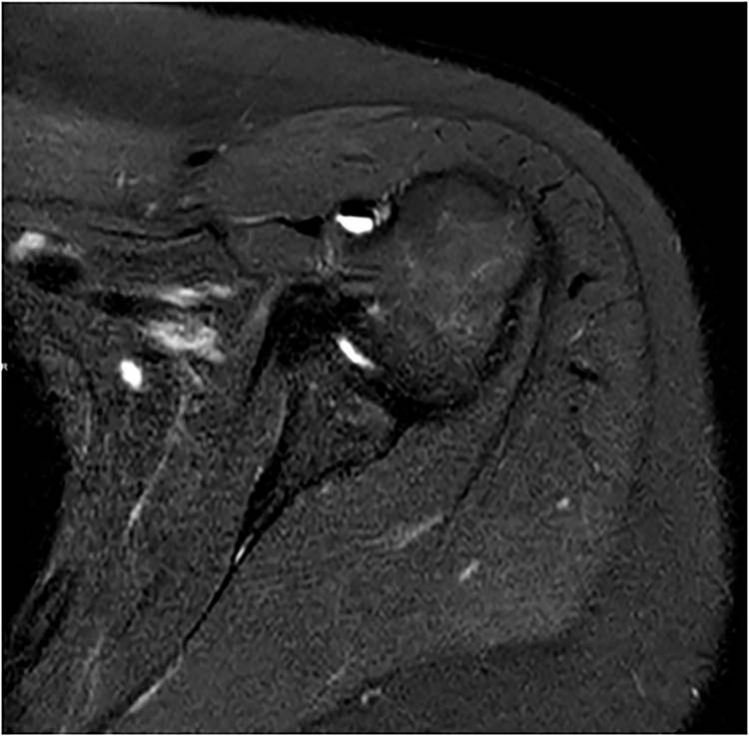
Coronal and axial T2 fat saturated image of left shoulder showing thickened inferior capsule of low T2 signal, characteristic of freezing phase in adhesive capsulitis.

**Figure 2:**
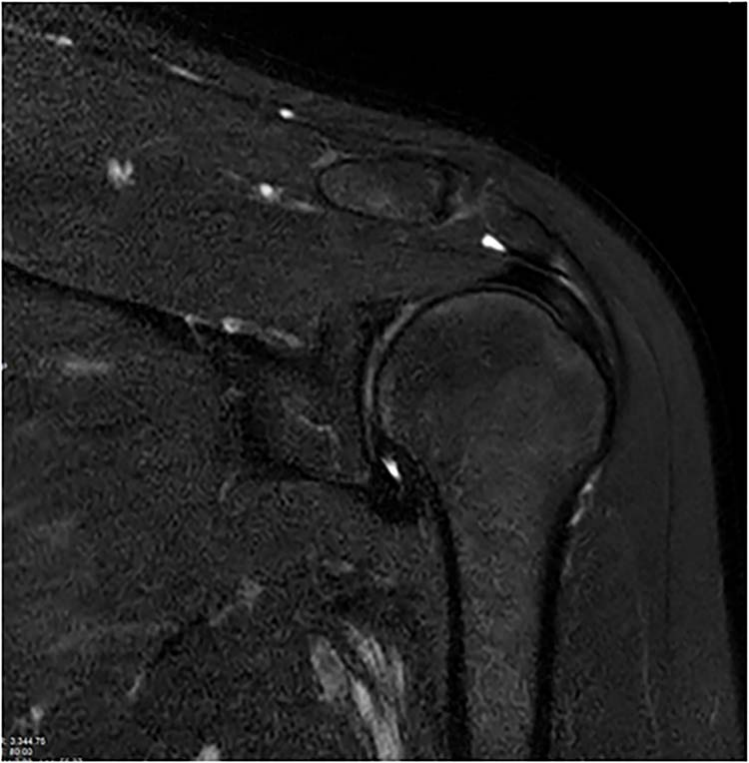
Coronal and axial T2 fat saturated image of left shoulder showing thickened inferior capsule of low T2 signal, characteristic of freezing phase in adhesive capsulitis.

Standard anterior/posterior and lateral X-rays of the affected shoulder were done. The patient was diagnosed with adhesive capsulitis. She was therefore referred to office-based extensive rehabilitation program to improve range of motion and strengthen shoulder muscles. Though MRI does not play a rule in the diagnosis and management, we opted to do one, after consenting the patient, trying to understand the peculiarity of the case (Figs 1 and 2).

Coronal and axial T2 fat saturated image of left shoulder showing thickened inferior capsule of low T2 signal, characteristic of the freezing phase in adhesive capsulitis.

## Discussion

The rise of COVID-19 as a global pandemic has created several health challenges. Most importantly, access to care and continuity of management has been hindered, particularly in countries where complete lockdowns have been implemented. The impact of COVID-19 on the orthopedic field has been affected in several ways, including significant decline in number of surgeries, clinic encounters, and ER visits as evident by Hsu *et al.* recent literature review [5]. Moreover, the rapid increase of SIRVA appended further challenges.

SIRVA is defined as shoulder pain arising within 48 h following the injection of a vaccine and lasting for 1 week. Interestingly, it has not yet been attributed to an organic cause [6]. The most common reported pathological processes of SIRVA in the literature were adhesive capsulitis and subacromial bursitis report [1].

Bass *et al.* performed a literature review examining further characteristic of shoulder injuries in 305 cases [1]. Females were found to be more affected with a peak age ranging between 50 and 70 years. The onset of symptoms varied depending on the type of vaccine administered. Viral vectors vaccines had a higher chance of immediate onset of symptoms (>24 h) compared to variable onset of symptoms for inactivated and mRNA vaccines.

Other studies reported similar findings, Biglia *et al.* reported similar cases of adhesive capsulitis that failed conservative management and were treated with ultrasound guided hydrodilatation [7]. Chu conducted a retrospective study of all patients presented to chiropractic, orthopedic, and physiotherapy clinics within 1 year following COVID-19 pandemic, and found 730 patients presented with shoulder pain following vaccinations, of which, 16 patients were diagnosed with adhesive capsulitis [8]. Findings were also reproducible in other studies [2, 9–12].

The main etiology of SIRVA has been attributed to suboptimal injection technique. This is supported by multiple studied reporting SIRVA cases after administration of influenza vaccination [4, 13]. Although multiple evidence based protocols were created for safe vaccine administration, these were not been standardized nor implemented across the globe [14].

Lastly, health care providers’ knowledge and awareness in the safe protocol of vaccination administration, adverse effects, and complications are still lacking. Mackenzie *et al.* found poor knowledge of SIRVA and the related anatomy in 42% and 55% of the practitioners, respectively [15].

In conclusion, SIRVA is a growing challenge for healthcare providers across the world, a standardized definition, implementation of safe vaccines injection protocols, and further education and awareness of SIRVA is needed to healthcare practitioners to allow better understanding and prevention.

## Data Availability

All data are available upon request.
